# Biosonar resolving power: echo-acoustic perception of surface structures in the submillimeter range

**DOI:** 10.3389/fphys.2014.00064

**Published:** 2014-02-26

**Authors:** Ralph Simon, Mirjam Knörnschild, Marco Tschapka, Annkathrin Schneider, Nadine Passauer, Elisabeth K. V. Kalko, Otto von Helversen

**Affiliations:** ^1^Department of Sensor Technology, Friedrich-Alexander University Erlangen-NurembergErlangen, Germany; ^2^Institute of Experimental Ecology, University of UlmUlm, Germany; ^3^Smithsonian Tropical Research InstituteBalboa, Panama; ^4^Institute of Zoology, Friedrich-Alexander University Erlangen-NurembergErlangen, Germany

**Keywords:** echolocation, bat, resolving power, structure recognition, resolution

## Abstract

The minimum distance for which two points still can be separated from each other defines the resolving power of a visual system. In an echo-acoustic context, the resolving power is usually measured as the smallest perceivable distance of two reflecting surfaces on the range axis and is found to be around half a millimeter for bats employing frequency modulated (FM) echolocation calls. Only few studies measured such thresholds with physical objects, most often bats were trained on virtual echoes i.e., echoes generated and played back by a computer; moreover, bats were sitting while they received the stimuli. In these studies differences in structure depth between 200 and 340 μm were found. However, these low thresholds were never verified for free-flying bats and real physical objects. Here, we show behavioral evidence that the echo-acoustic resolving power for surface structures in fact can be as low as measured for computer generated echoes and even lower, sometimes below 100 μm. We found this exceptional fine discrimination ability only when one of the targets showed spectral interferences in the frequency range of the bats′ echolocation call while the other target did not. This result indicates that surface structure is likely to be perceived as a spectral quality rather than being perceived strictly in the time domain. Further, it points out that sonar resolving power directly depends on the highest frequency/shortest wavelength of the signal employed.

## Introduction

The angular resolution of visual systems is much finer than that of biosonar systems because of the much shorter wavelength of light; however, echolocation has distinct benefits over visual perception, especially when perceiving small objects (Boonman et al., 2013) and perception of depth, i.e., resolution on the range axis is much finer than in vision. Visual recognition of surface structure or structure depth is difficult, not particularly fine and depends on the distance (e.g., cats can resolve about 1 cm in 50 cm distance; Blake and Hirsch, 1975). In contrast, biosonar systems are particularly suited for this task as the distance of objects is directly encoded in echo delay. Resolving power on the range axis or echo delay resolution were primarily investigated by training bats that used frequency modulated (FM) echolocation signals to distinguish between plates with holes of different depths, thus representing two reflecting surfaces (two-front objects) with a particular distance. These early experiments showed that bats (*Eptesicus fuscus* and *Myotis myotis*) are able to distinguish differences in hole depth when they exceeded 0.6–1.0 mm, depending on the absolute hole depth, which varied around 4–8 mm (Simmons et al., 1974; Habersetzer and Vogler, 1983). There were also some reports on discrimination of sandpaper (Zagaeski and Moss, 1994) and studies of discrimination of structured pearls (Falk et al., 2010) where discrimination thresholds down to 0.5 mm were observed for some animals, while others needed differences more than 1.5 mm. Psychophysical studies, which investigated echo-acoustic resolution of bats using computer-generated echoes revealed that bats were able to distinguish virtual depth differences as low as 200 μm. In those experiments, positive and negative target had a virtual depth around 1.3 mm, which was simulated by playing back two copies of the bat's echolocation call with a certain delay (*Megaderma lyra*, Schmidt, 1992). As both targets had a structure depth, which would be recognizable for the bats, in this study the threshold for discrimination of two differently structured objects was measured. However, a more adequate measure to determine the resolving power of a sonar system is the threshold for which an object is judged as “structured” vs. “smooth” and may be called structure recognition threshold (Mogdans et al., 1993; Simmons et al., 1998). Studies obtaining this threshold also with computer generated echoes found values between 2 mm and 340 μm (Simmons et al., 1990, 1998; Mogdans et al., 1993). The latter threshold translates to a temporal separation of only 2 μs and this well-designed experiment showed that the two reflecting surfaces of those targets were indeed separately recognized by their absolute delay (Simmons et al., 1998).

The most obvious echo features allowing such fine discrimination are spectral interferences caused by the surface structure (Habersetzer and Vogler, 1983; Schmidt, 1988; Simmons et al., 1998; Simon et al., 2006). The smaller a surface structure, the smaller is the wavelength (= the higher the frequency) that is cancelled. Therefore, the high-pitched broadband FM signals of many gleaning bats are particularly suited to recognize interferences and resolve structures (Siemers and Schnitzler, 2004; Simon et al., 2006; Yovel et al., 2011). Consequently, the structure recognition threshold directly depends on the smallest wavelength of the employed signal. Below this threshold, all structures should be judged smooth because the interferences appear at wavelengths smaller than the smallest wavelength of the bat's call and thus structural differences should not be perceived. Above this threshold, different structures should be recognized as structured because they all cause interferences in the reflected echo, making discrimination more difficult. Yet the highest discrimination ability should be found for pairs of targets where one causes interferences and is regarded as structured whereas the other does not and therefore is regarded as smooth.

To test these hypotheses, we trained free-flying nectar-feeding bats (Phyllostomidae: Glossophaginae: *Glossophaga soricina*) to discriminate between spheres with different surface structures starting from 63 up to 917 μm. We first determined the structure recognition threshold and then subsequently trained bats on different pairs of structured targets around this threshold. We compared the discrimination performance of the bats with echo acoustic properties of the targets to deduce the echo features used for discrimination. We used this nectar feeding bat species, because they belong to the guild called “narrow space passive/active gleaning bats” (Denzinger and Schnitzler, 2013). Members of this guild are characterized by their high-pitched, broadband echolocation signals, which they employ to find tiny flowers or fruits in front of vegetation and their signals should be particularly suited to resolve finest structures.

## Materials and methods

### Structured targets

We used celluloid spheres with a diameter of 40 mm (Type Basis, Tibhar Tibor Harangozo GmbH, Saarbrücken, Germany) as basic form for the targets. The advantage of using spheres as targets is that their echoes are nearly independent of the angle of sound incidence. To create targets with different surface structures, the spheres were covered with a monolayer of fine glass beads of different sizes (63–917 μm; SiLibeads^®^, Sigmund Lindner GmbH, Warmensteinach, Germany). We verified the sizes of the purchased glass beads by measuring 500 beads of every size under the microscope (see Figure 3A for sizes and SD). To coat the spheres with glass beads, the spheres were mounted on a pin plug. Then, aerosol fixative (Photo Mount™; 3M, St. Paul, Minnesota, USA) was applied thinly and evenly onto the spheres′ surface. Subsequently, the spheres were fixed with their pin plug in the center of a cap of a jar, which contained about 50 g of a certain size of glass beads. The jar was closed, which brought the sphere into the middle of the jar. We shook the jar until the spheres were covered with a monolayer of glass beads. The bead layer of each sphere was then surveyed for irregularities and holes; excess beads were removed with a fine brush. Spheres that were not uniformly covered with glass beads were discarded. See Figure 3A for magnified images of the targets′ surface.

### Behavioral experiments

**Figure 1:**
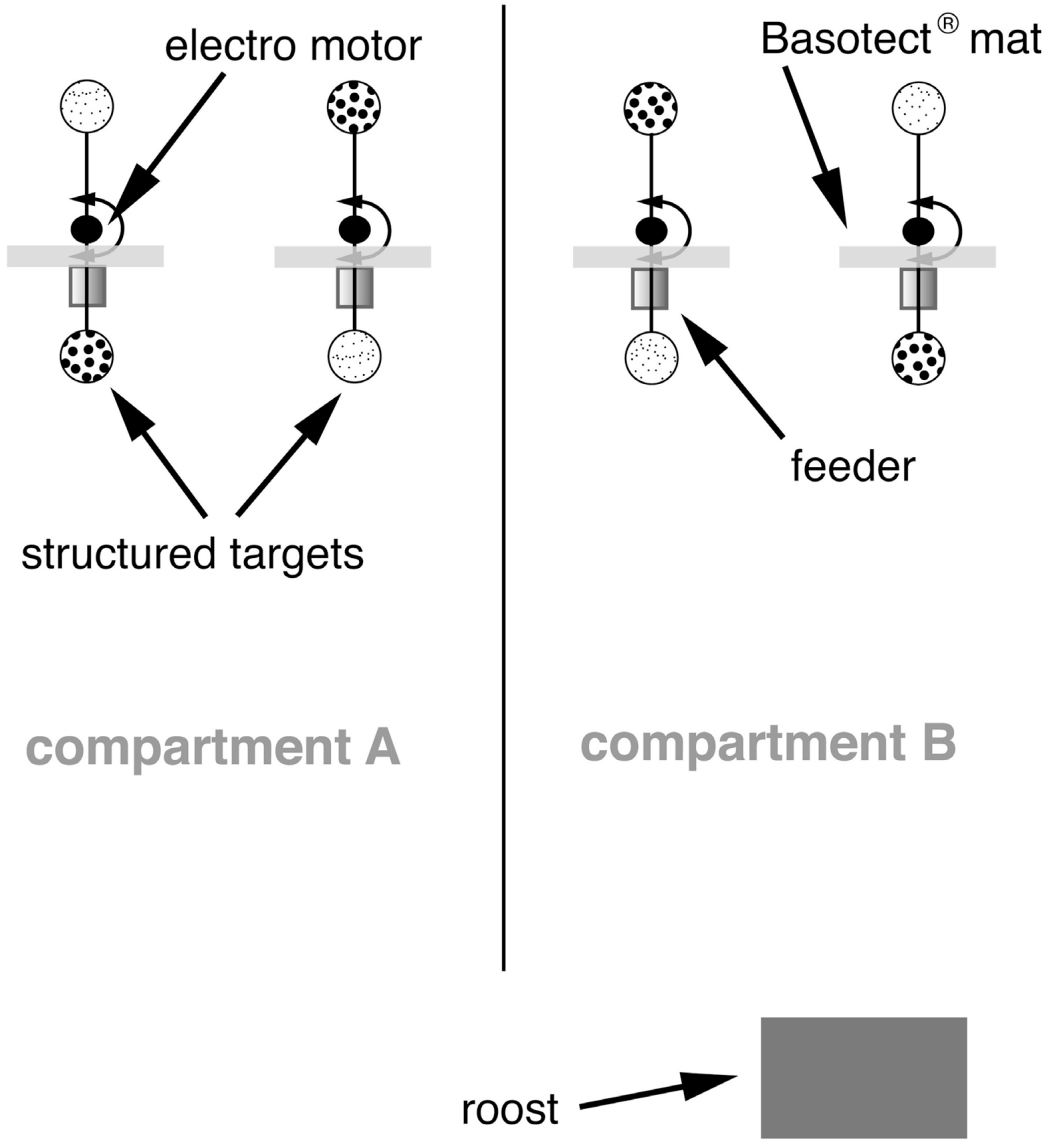
**Experimental room and training apparatus.** The room was divided into two compartments, which were connected at one end. Two feeders were placed at the other end of each compartment. Above each feeder, one of two different structured targets were presented. Targets could be replaced by an electro motor. The motor, valves and other targets that should not be presented were completely hidden behind sound-absorbing Basotect® mats.

Bats were trained using a “two alternative forced choice” paradigm to discriminate between two targets that differed in their structure. During the training, we successively decreased the difference in structure depth of the two targets, while the bats were always rewarded at the target with the smaller bead size. As a reward, small drops (10–20 μl) of sugar water (17% mass/mass; mixture of glucose, fructose and sucrose: 37:37:26, a mixture similar to the sugar composition of nectar of bat-pollinated flowers; Baker et al., 1998) were applied by magnetic pinch valves (Type 2/2–NC: Gr.1; ASCO Joucomatic, Ölbronn- Dürrn, Germany) into cylindrical feeders. To find out which of the two feeders obtained food, animals had to find the feeder displaying the target with the finer structure (Figure 1). At this feeder, the bats triggered an infrared beam sensor, which was integrated in the front opening of the feeder when inserting their snout. The experimental room (5 × 2.5 × 2.2 m; length × width × height) was divided into two compartments, which were connected and formed a U-shaped enclosure (Figure 1). At each of the ends of the two compartments, a pair of feeders presenting structured targets was placed and in each compartment only one feeder provided a reward. The structured targets were exchangeably presented above the feeders using a stepper motor (Sanyo Denki, Sanyo, Moriguchi, Osaka, Japan) or servomotors (Faulhaber GmbH & Co. KG, Schönaich, Germany), respectively, controlled by a computer. To avoid that bats were present when the targets were replaced, the motors changed the targets every time the bats were visiting the feeders in the respective other compartment. A pseudo-random sequence determined on which side the target, which provided a reward (positive stimulus) was presented (see Figure 1). Bats were rewarded only after they interrupted the infrared light beam of the feeder with the positive stimulus. When the bat chose the negative stimulus, none of the feeders in the respective compartment gave reward, but the bat had to go to the other compartment to get rewarded again. Thus, the bats were forced to alternate between the two compartments to get food and they usually made between 1000 and 1400 decisions each night in each compartment. Bats were under a 12 h/12 h dark/light regime. All data acquisition was solely made throughout the dark phase.

During two experimental series, a total of six bats, adult males of the species *Glossophaga soricina*, were trained to discriminate between targets covered with glass beads of different diameters. The rewarded target was always the target with the finer structure.

#### Experiment I

To measure the structure recognition threshold, we first accustomed the bats (*n* = 6) to the task by initial training with a pair of structured targets that showed the greatest possible difference in mean bead diameter (63 vs. 917 μm). As soon as the discrimination performance reached a stable level (usually 2–3 days), the unrewarded object was exchanged by an object that had a finer structure. From this experiment, we deduced the structure recognition threshold, which we defined as 75% correct decisions in the discrimination task (see Figure 4A).

#### Experiment II

We trained four bats on different pairs of structured targets, all having a similar difference in structure depth. One pair had two targets that both were beneath the threshold deduced in Experiment I, which was pair A (63 vs. 260 μm; difference: 197 μm), then pair B (350 vs. 517 μm; difference: 167 μm) which had one target above and one target beneath the threshold and pair C which had targets both with structures above the threshold (702 vs. 917 μm; difference 215 μm). As we recognized that pair B was easily discriminated, we also introduce pair B′ (350 vs. 411 μm; difference: 61 μm). The bats were always rewarded at the smaller beat size and were trained with the pairs A, B, B′, and C for two nights. The data for pair A were deduced from experiment I because there the bats were already trained on this particular pair. To train the bats on Pair B, we carefully accustomed them by training them on 260 vs. 917 μm first and then on 260 vs. 702 μm. After they learned to discriminate both pairs, we confronted them with the pair B for two nights and then with then pair B′ for two nights. To familiarize them with discrimination of coarser objects like pair C, we trained them on 411 vs. 917 μm, then 517 vs. 917 μm to finally confront them with the pair C.

The bats decisions were analyzed in blocks of 100 decisions. At the beginning of every night, the bats needed some initial training to reach the discrimination levels from the night before; therefore, we discarded the first 500 decisions of each night for each compartment. Moreover, we discarded single blocks of 100 decisions when the proportion of correct choices (%) differed more than 15% in between the two compartments, as this indicated that the bats were not focusing on the task. All remaining decisions were included in the final analysis and we calculated mean values per bat and target pair. To test whether the bats′ decisions differed according to the presented target pair, we conducted a general linear mixed model (GLMM) with percentage of correct choices as dependent variable, target pair as fixed factor and bat ID as random factor. Subsequent *post-hoc* tests were conducted with a Bonferroni correction. We used a Shapiro-Wilk test to ascertain that residuals did not deviate significantly from a normal distribution. The GLMM was calculated using SPSS version 20 (IBM Corporation, New York, USA).

We made recordings of the echolocation calls of three bats (*Glossophaga soricina*, one adult male, two adult females) flying toward a feeder. We used a 1/8″ microphone, with preamplifier (Brüel & Kjaer, Nærum, Denmark), which was connected to the power module 12AA (G.R.A.S. Sound & Vibration, Holte, Denmark). We digitized the microphone-signal with an UltraSoundGate 116 Hm (Avisoft Bioacoustics, Berlin, Germany) at a sampling rate of 1000 kHz with 16 bit resolution. Calls were analyzed with MATLAB (The MathWorks, Natick, Massachusetts, USA).

### Echo-acoustic measurements

The echoes of structured targets were measured by ensonifying them in a distance of 20 cm with a continuously replayed MLS Signal (Maximum Length Sequence) of 16 383 samples length. We recorded the reflected signal and obtained the impulse responses (IR) by deconvolution of the reflected echo and the original MLS. The IR were analyzed regarding their maximum amplitude, their duration, their roughness and spectra. For each structured target, we analyzed IR sampled from 51 different directions (acquired over an angle of 90° in 1.8° steps). To compare maximum amplitudes of different targets, we determined the maximum amplitude for each of the measurements from different directions and then determined the mean maximum amplitude. The duration of the IRs was measured using an amplitude threshold (mV) that had been determined for each target: we chose 20% of the mean maximum amplitude of the respective target. Using this threshold, we determined start- and end-point for each IR. As a measure of IR roughness we used the base 10 logarithm of the fourth moment (log_10_M4) as published by Grunwald et al. (2004). Power spectral density of the IR was calculated for a rectangular window of 1024 samples. The resulting spectra were corrected for the frequency response of the speaker; the ±6 dB cut-off frequency limited the bandwidth of the system from 40 and 160 kHz. This method is described in detail in earlier work (Von Helversen et al., 2003; Simon et al., 2006).

### Theoretical considerations on the echo of structured targets

When sound impinges on the surface of a smooth sphere, there are basically two ways of reflection (i) On the lateral areas of the sphere, the sound is scattered and reflected away from the sound source and is not part of the reflected echo (ii) At the area of the sphere which is closest to the sound source and where the tangent is perpendicular to sound propagation, the sound is reflected back to the sound source. The size of this area and thus the reflected sound energy, which refers to the acoustic cross section, depends on the size of the sphere. If the sphere is smaller than the wavelength of the employed signal, it has to be considered in the Rayleigh domain. Then reflected energy not only depends on the size of the sphere but also on the wavelength of sound (Yovel et al., 2011). If the sphere is much larger than the wavelength, reflected energy is equal for all wavelengths and only depends on the size of the sphere. As we used the same size of spheres that were much larger than the wavelength throughout the experiments, the area and the reflected energy theoretically should be the same. However, as this spheres were covered with spherical beads that are smaller than the wavelength of the signal, we had an object with many Rayleigh scatterers (Yovel et al., 2011). This means that reflected energy should also depend on the size of the structure beads and because of interference, sound with a wavelength (λ_cancelled_) that is four times the structure depth (*d*) is cancelled:

(1) $$\lambda_{\text{cancelled}}=4d,$$

As the wavelength (λ) is the quotient of speed of sound (c) and the frequency (ν), spectral notches will occur at frequencies:

(2) $$\nu_{\text{notch}}=\frac{c}{4d},$$

To give an example: The largest glass beads that we used had a mean size of 0.92 mm; thus, for a wavelength of 3.67 mm cancellation was expected, which translates into a frequency of 93 kHz where we expect cancellation or a spectral notch, respectively.

Moreover, the surface structure provides different levels where the sound can be reflected and in the reflected echo those reflections result in amplitude peaks, which are separated in time. The main reflection should happen on the upper surface of the structured beats and the second one on surface of the sphere. In the reflected echo, the two main amplitude peaks should be separated by the time the sound needs to travel twice the distance of the structure depth. Assuming a speed of sound of 340 m/s (or 340 μm/μs), this time gap (t_gap_) between the main amplitude peaks would be:

(3) $$t_{\text{gap}}=\frac{2d}{340},$$

As the objects used in the experiments had structure depths of approximately 50 μm to 1 mm, we expected amplitude peaks in the echoes with separations from 0.4 to 6 μs.

## Results

### Echo-acoustic properties of the structured targets

**Figure 2:**
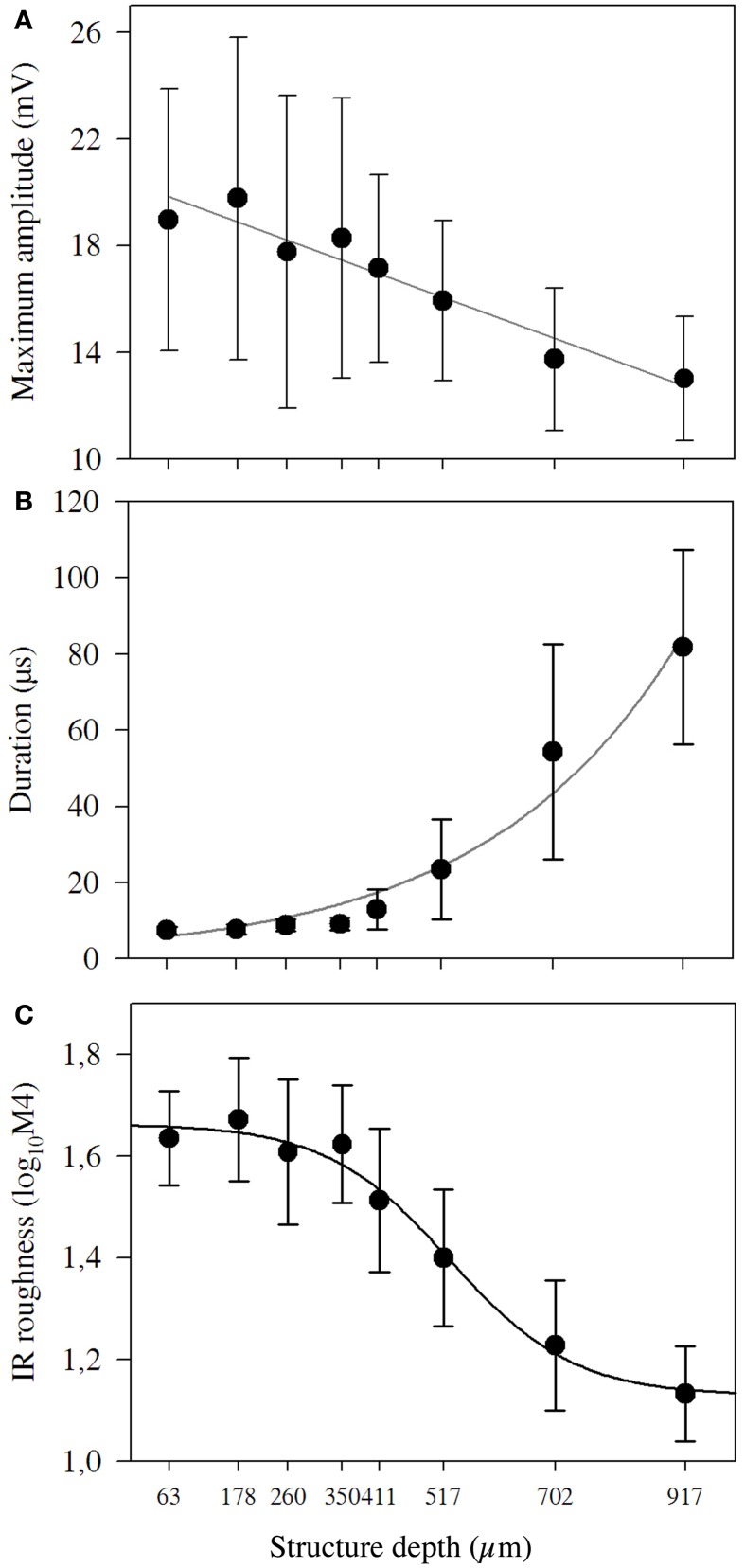
**Amplitude, duration and roughness of impulse responses of structured targets.** For each target, we analyzed 51 impulse responses measured from different directions. **(A)** Mean maximum amplitude and standard deviation of the targets′ impulse response. The black line marks the linear regression (*y* = 0.68 − 0.00028x; *r*^2^ = 92.2; *p* < 0.001). **(B)** Mean duration and standard deviation of the targets′ duration. The black line marks the exponential growth regression (*y* = 4.8e 0.003x; *r*^2^ = 98.3; *p* < 0.01). **(C)** Mean IR roughness and standard deviation (log_10_M4) for targets with different structure depths. The black line marks the sigmoidal regression.

**Figure 3:**
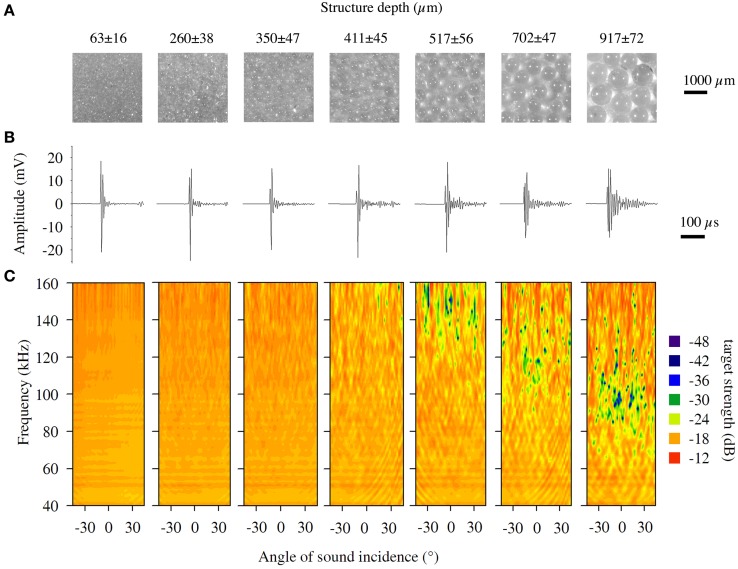
**Magnified images, impulse responses and spectral directional plots for structured targets. (A)** Magnified images (six times) of the structured targets, which were spheres coated with fine glass beads. **(B)** Representative impulse responses and **(C)** spectral directional plots of the structured targets.

The echo-acoustic properties of the targets such as maximum amplitude, duration, and roughness depended on structure depth (Figure 2). Maximum amplitude decreased linearly and showed high variability when measured from different directions (Figure 2A). The duration of the IR exponentially increased (*y* = 4.8e 0.003x; *R*2 = 98.3; *p* < 0.01; Figure 2B) for coatings larger than 411 μm and it increased to levels much higher than we expected due to the structural depth of the coating. The largest duration measured was around 80 μs, which represents a structural depth of 15 mm. Obviously for coarser coatings, sound was reflected at lateral parts of the sphere and multiple reflections within the bead layer contributed to the prolongation. IR roughness varied between 1.1 and 1.6 log_10_4M and showed a sigmoid declining curve with the steepest part between 411 and 702 μm. The increasing length and the decreasing amplitude and roughness are also obvious in the IRs exemplarily shown in Figure 3B.

Spectral target strength of the structured targets′ echoes varied around −20 dB and the sizes 63, 260, and 350 μm showed low variance for recordings from different directions as well as low variation for the whole frequency area (Figure 3C). First sporadic spectral notches occurred for the target covered with glass beads of 411 μm diameter, and first spectral bands where cancellations occur more frequently are visible for the target 517 μm around 150 kHz. With increasing structural depth, these cancellations occurred also at lower frequencies. For the object with the 917 μm coating, these notches occurred around 90 kHz, which is exactly the frequency area where we would expect cancellations due to the structure depth of the coating (Figure 3C). Since the beads had a certain variation (mean *SD* ± 50 μm), the cancelled frequency band was not sharp but somewhat blurred.

### Results of the behavioral experiments

**Figure 4:**
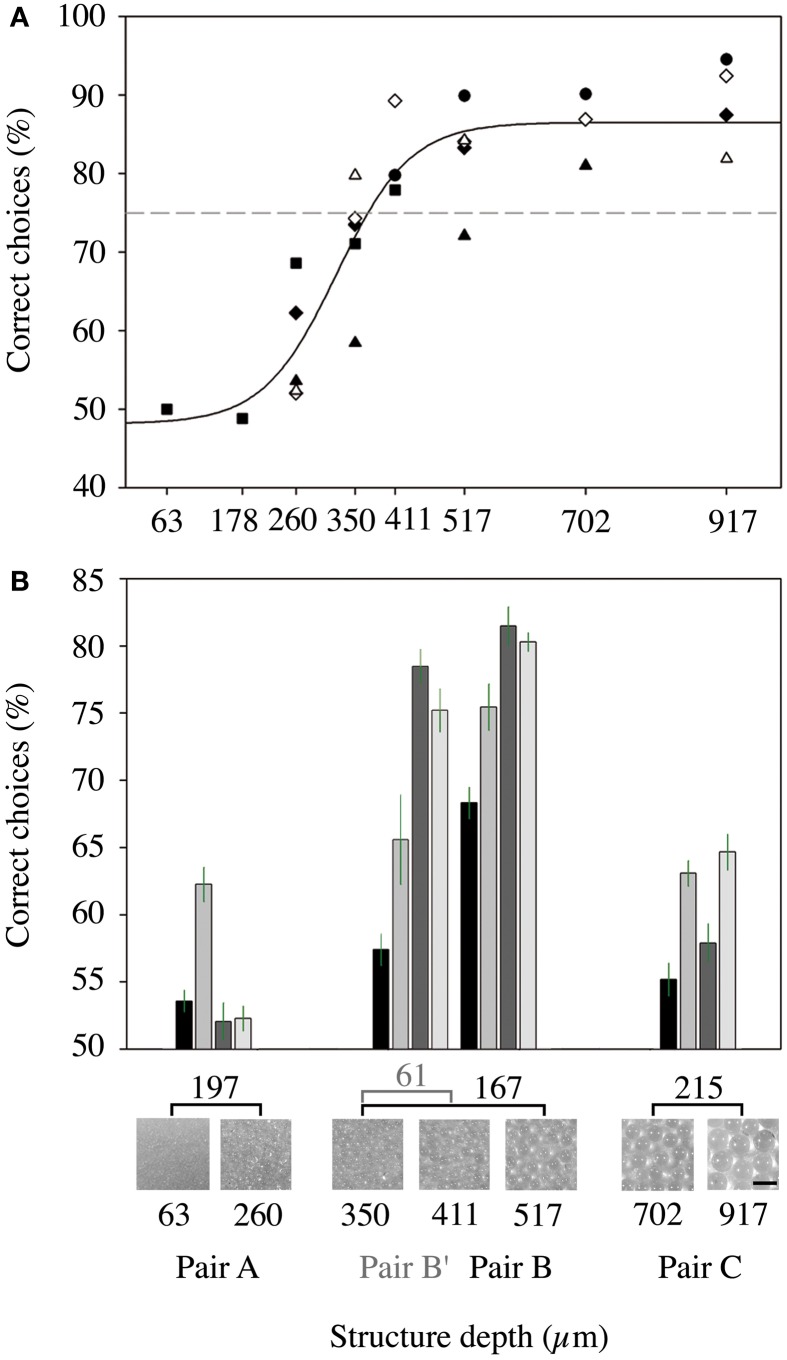
**Results of the behavioral experiments with *Glossophaga soricina*. (A)** Psychometric function of the ability of six bats to discriminate between targets with different structure depth (experiment I). The results of the different bats are marked by different symbols. The x-axis gives the structure depth of the unrewarded target, while we always trained on the target with the 63 μm structure. The dashed line marks the 75% threshold. **(B)** Results of experiment II. Mean percentages of correct choices for discrimination of selected pairs of targets. Each pair was presented to each of the four bats for two nights. The error bars indicate the standard error. X-Axis: upper line: difference of structure depth of the respective pair. Middle line: magnified images of the structured targets. Two bottom lines: structure depth of the targets in μm and name of the respective pair.

In experiment I, we determined the threshold for which a structure is recognized vs. a smooth surface for the nectar-feeding bat species *Glossophaga soricina* by training six individual males on pairs of structured targets. The depth of the structure was converged while the bats were trained to get a reward at the smoothest sphere (covered with the finest bead size of 63 μm). We found that the percentage of correct choices started to decrease when an unrewarded object of less than 500 μm was presented (Figure 4A) and fell below 75% for unrewarded targets smaller than 380 μm (see intercept point with the 75% threshold in Figure 4A). Thus, we can deduce a smallest resolvable structure of 380 μm for *G. soricina*. Below this threshold, all targets should be regarded as unstructured or smooth.

We tested this assumption in experiment II, where we trained bats (*n* = 4) to discriminate between different pairs of structured targets. The pair with targets 63 vs. 260 μm (pair A) both had coatings that were smaller than the threshold. Only one animal was able to reach a level of 62.3% correct choices while the other animals reached only around 53%, (55.0 ± 4.9%; mean ± *SD*, *n* = 4 bats with a mean of 2375 ± 818 decisions) suggesting that all bats made their decision randomly (Figure 4B). The pair of targets that both had coatings above the threshold (pair C; 702 vs. 917 μm) was discriminated at a slightly higher level with 60.2 ± 4.4% correct choices (mean ± *SD*, *n* = 4 bats with a mean of 2400 ± 365 decisions) but no bat reached 75% or more (Figure 4B).

We also offered a pair that had mixed targets, one with structures greater than the threshold of 380 μm and one with structures smaller than 380 μm (pair B; 350 vs. 517 μm). For this combination, three bats surpassed the 75% threshold and on average the animals made 76.3 ± 6.0% correct choices (mean ± *SD*, *n* = 4 bats with a mean of 2275 ± 921 decisions, Figure 4B). Each bat discriminated the targets within this pair significantly better than the targets within pairs A and C [GLMM: *F*_(3, 9)_ = 11.665, *p* = 0.002; pairwise comparisons: pair A vs. pair B, *p* = 0.002; pair C vs. pair B, *p* = 0.015, pair A vs. pair C, *p* = 0.999]. Even if the bead size difference was further decreased by presenting pair B′ (350 vs. 411 μm, Figure 4B) and the difference in structure depth was only 61 μm, the level of correct choices (69.2 ± 9.6%; mean ± *SD*, *n* = 4 bats with a mean of 2750 ± 1445 decisions) was still significantly higher than for pair A and somewhat, but not significantly, higher than for pair C [GLMM: *F*_(3, 9)_ = 11.665, *p* = 0.002; pairwise comparisons: pair A vs. pair B′, *p* = 0.034; pair C vs. pair B′, *p* = 0.287, pair B vs. pair B′, *p* = 0.592].

One animal performed poorer than the others in Experiment I (Figure 4A) and also in Experiment II (Figure 4B). This is not necessarily a matter of intelligence but just an indication that this animal used another strategy—it may have chosen a more exploratory strategy rather than a very efficient strategy. Even if they did not pay attention to the task, they were rewarded in 50% of the cases.

### Ethical standards

Experiments were approved by the University of Erlangen-Nuremberg, adhered to the ASAB/ABS Guidelines for the Use of Animals in Research, and were in compliance with the current laws of Germany. Throughout the experiments, the animals were regularly checked regarding their weight and state of health.

## Discussion

**Figure 5:**
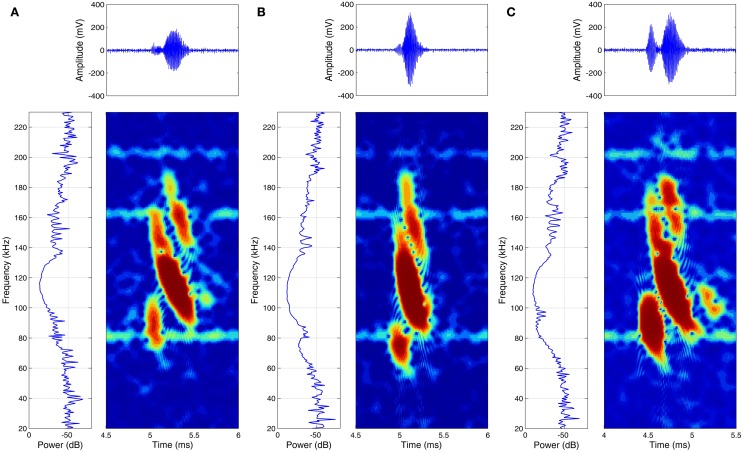
**Examples of high-pitched echolocation calls of three specimens of *Glossophaga soricina* while approaching a feeder.** For each specimen **(A–C)** the power spectrum (left) the spectrogram (right) and the time signal (top) is given.

We found that *Glossophaga soricina* recognized surface structures when they are coarser than 380 μm. This supports the threshold of 340 μm found in studies where computer generated echoes were used (Simmons et al., 1990, 1998) and shows that also physical objects with such minute structures can be perceived by the bats even in flight. As all targets beneath this threshold were not distinguished by the bats, they all must have been perceived as equally smooth surfaces, which is supported by the fact that in experiment II for pair A (63 vs. 260 μm) discrimination levels were particularly low. The resolvable structure depth should directly depend on the highest frequency/smallest wavelength of the bats′ calls. *Glossophaga soricina* has a short (0.3–1.3 ms) FM call with two harmonics (1st 95–55 kHz; 2nd 150–86 kHz; Goerlitz et al., 2010; Knörnschild et al., 2010) and in own recordings we also observed a third harmonic starting 190 kHz down to approximately 140 kHz (see Figure 5). Due to Formula I we expect structures of 450 μm (exactly 447 μm) causing notches at a frequency of 190 kHz (wavelength of 1.8 mm). This fits quite well with the results from experiment I. Here a structure depth of 411 μm was the smallest structure, which was recognized vs. a smooth surface and somewhat higher discrimination levels were reached for the 517 μm structure. Interestingly, those structures were not only recognized vs. a very smooth surface but also vs. a target with a structure of 350 μm (Experiment II), providing only a minimal differences in structure depth (167 and 61 μm, respectively). What echo feature gives the best explanation for this particularly fine discrimination performance? Basically there are three different echo features to consider: temporal features, spectral cues, and intensity cues. None of them are in principle mutual exclusive, but we want to discuss them separately.

As a target with a coarse surface reflects less energy than a target with a finer surface structure (Yovel et al., 2011), differences in intensity could be a simple cue allowing structure discrimination. Indeed, we found that the maximum amplitude linearly decreased with increasing structure depth. If we compare the differences of the maximum amplitude of the structures used in experiment II and translate them into differences of echo intensity (dB), pair B showed the highest difference (1.2 dB), whereas the other pairs showed lower values (pair A: 0.5, pair C: 0.6 dB, pair B′: 0.6 dB). As pair B was discriminated best, intensity based structure recognition would explain our behavioral results. But because a value of 1.2 dB is on the lower limit of recognizable intensity differences (1–3 dB; Airapetianz and Konstantinov, 1974; Simmons et al., 1974; Schnitzler and Henson Jr, 1980), the standard deviation was high and because the bats made their decision in flight it is unlikely that the bats used amplitude or intensity differences alone to discriminate between the different surface structures.

A structured surface provides different planes of reflection and therefore causes amplitude peaks or intervals within the reflected echo, which are separated by time as defined by formula 3 (see chapter theoretical considerations). In the case of the structured targets used in the experiments, this time delay should theoretically be between 0.4 and 6 μs, which is particularly short to recognize with a call that is about 1 ms long. However, if the bats would process the auditory information in a kind of cross-correlation of call and echo, they would receive a sharp IR with distinct amplitude peaks and would thus be able to resolve surface structure in the time domain. In this case, they would not necessarily need to employ a signal that has a wavelength short enough to resolve the surface structure. They would simply need a signal providing some bandwidth to sharpen the deduced IR. It is not clear whether bats have IR representation through cross-correlation of signal and echo (Schnitzler and Henson Jr, 1980; Wiegrebe, 2008); however, we have to consider temporal cues in the IRs of the structured spheres, as some studies suggest that there might be something similar available to bats (Simmons, 1989; Saillant et al., 1993; Simmons et al., 1998).

Due to the nature of the structured spheres, with densely packed glass beads causing many reflections, it was not possible to deduce single separated amplitude peaks in the IR of the objects. However, as amplitude peaks separated by time would also prolong the echo, we deduced absolute length of the IR as a temporal measure influenced by the surface structure. The IR duration predictably changed with structure depth, as seen in Figures 2B, 3B. but were considerably longer than expected only due to effects because of the structure depth. The reason might be that multiple reflections within the surface structure were likely to happen especially for coarser structures. The pairs of objects, presented in experiment II, showed absolute durations between 7.4 and 81.7 μs. If the bats based their decision on temporal differences in the IRs they had to detect a differences of only 1.4 μs for pair A, followed by pair B with 14.3 μs and pair C with 27.5 μs. Thus, assuming a temporal based discrimination mechanism, the bats should have performed best with discrimination of pair C as it showed the highest difference in IR-duration. However, as the bats performed significantly better for the discrimination of pair B (14.3 μs), it is difficult to explain the results of the behavioral experiments by the temporal differences found in the echoes of the structured targets.

Another temporal echo feature, which is discussed to be used for the classification of natural textures, is IR roughness (Grunwald et al., 2004). We used the base 10 logarithm of the fourth moment (log_10_M4) as a measure of IR roughness and we found a sigmoidal relation between structure depth and IR roughness, with a maximum of 1.7 and a minimum of 1.1 log_10_M4. As the steepest part of the sigmoid curve is between the targets 350 and 702 μm, discrimination based on IR roughness could explain the results of both experiments. However, the IRs of our targets were very short—IRs for which discrimination and classification was shown were much longer, Grunwald et al. (2004) used IRs of 16.4 ms length—making recognition of IR roughness implausible. Moreover, in those experiments a minimum difference of 0.75 log_10_M4 was needed for correct discrimination and that is more than the total range of all our targets, which was 0.6 log_10_M4. For this reasons it is unlikely that the bats discriminated based on differences in IR roughness.

Many studies showed before that bats are able to perceive spectral interference patterns (Bradbury, 1970; Simmons et al., 1974; Schmidt, 1988, 1992) and also the results of this study are pointing to a spectral based perception of surface structure. Spectral based discrimination of fine surface structures has the distinct advantage that small differences in structure depth will result in drastic changes of echo spectra, because of the hyperbolic relationship between structure depth and spectral interferences (see formula 2 in theoretical considerations). In experiment II a pair of structured targets with a difference of only 61 μm (Pair B′) could be distinguished significantly better than a pair of objects providing a difference of 197 μm (Pair A). The simplest and most parsimonious explanation for this performance would be that the bats recognized structure in terms of spectral interferences (or at least in terms of a slight low pass filter effect), in the third harmonic of their calls (Figure 5), when they received echoes of the 411 μm structure (Figure 3C), while they received a unfiltered echo reflected by the 350 μm structure (Figure 3C), which would be therefore regarded as smooth. These two targets, even if they have a minimal difference in structure depth and temporal differences are extremely limited, they are particularly easy for the bats to discriminate because they have very obvious spectral differences.

In conclusion, it is important to mention that bats will of course benefit from all differences and all echo information accessible to them. Especially when they fly around in their natural environment, where they have to orient in complex surroundings and solve challenging foraging tasks precise acoustic images are crucial. A fine echo-acoustic resolution may for example help bats to find roosts and structures they can cling on to, it may facilitate insectivorous gleaning bats to detect silent insects perching on leaves (Geipel et al., 2013) and it may help to classify prey. In case of nectar-feeding bats it may facilitate recognition of floral signals (Von Helversen and Von Helversen, 1999; Simon et al., 2011) or detection of small structures on the flowers′ surface, which help to get to the nectar.

### Conflict of interest statement

The authors declare that the research was conducted in the absence of any commercial or financial relationships that could be construed as a potential conflict of interest.
